# Small angle X-ray scattering data and structure factor fitting for the study of the quaternary structure of the spermidine N-acetyltransferase SpeG

**DOI:** 10.1016/j.dib.2015.11.044

**Published:** 2015-11-30

**Authors:** Steven Weigand, Ekaterina V. Filippova, Olga Kiryukhina, Wayne F. Anderson

**Affiliations:** a DND-CAT Synchrotron Research Center, Argonne National Laboratory, Argonne, IL 60439, USA; b Center for Structural Genomics of Infectious Diseases, Northwestern University, Feinberg School of Medicine, Department of Biochemistry and Molecular Genetics, Chicago, IL 60611, USA

## Abstract

Here we describe the treatment of the small-angle X-ray Scattering (SAXS) data used during SpeG quaternary structure study as part of the research article “Substrate induced allosteric change in the quaternary structure of the spermidine N-acetyltransferase SpeG” published in Journal of Molecular Biology [1]. These data were collected on two separate area detectors as separate dilution series of the SpeG and the SpeG with spermine samples along with data from their companion buffers. The data were radially integrated, corrected for incident beam variation, and scaled to absolute units. After subtraction of volume-fraction scaled buffer scattering and division by the SpeG concentration, multiple scattering curves free of an inter-molecular structure factor were derived from the dilution series. Rather than extrapolating to infinite dilution, the structure factor contribution was estimated by fitting to the full set of data provided by dividing the scattering curves of a dilution series by the curve from the most dilute sample in that series.

## **Specifications table**

**Table:** 

Subject area	Biology
More specific subject area	Quaternary protein structure analysis
Type of data	Text file, figure
How data was acquired	Small-angle X-ray scattering
Data format	Raw, analyzed
Experimental factors	Sample preparation and dilution series as described in Filippova et al., 2015 [1]
Experimental features	Dilution series data collection of SpeG in the presence and absence of spermine at a synchrotron SAXS beamline and derivation of multiple structure factor-free absolute scattering curves.
Data source location	Sector 5, Experimental hutch 5-IDD, Advanced Photon Source, Argonne IL, United States
Data accessibility	Data is with this article

## **Value of the data**

• Providing the data while also describing how they were collected and treated facilitates the data collection of other researches attempting to follow the same technique.
• Detailing the data used in small angle scattering analysis provides a means for other researchers to evaluate future methods of structural interpretation from these data.

## 1 Data

These data were collected as described in detail in [1]. The data include small angle X-ray scattering curves from two sets of a two-fold dilution series containing five sample dilutions. One series was the spermidine N-acetyltransferase SpeG in the absence of spermine, and the other was SpeG in the presence of spremine. The data were collected on two CCD based area detectors, which were then radially integrated to provide intensity versus momentum transfer vector scattering curves (*I* vs 4πsinθ/λ). These were corrected for incident beam and transmission variation by division of the measured relative transmitted beam intensity on a diode in the beam stop. Next the data were scaled to absolute units by comparison to the small angle scattering of water in the same system.

Scattering curves of empty capillary exposures were subtracted from those of the protein dilutions and the buffer alone. The resultant buffer scattering, scaled to one minus the expected protein volume fraction, was subtracted from the protein dilution scattering curves. These data were then divided by the SpeG concentration. In the case of SpeG alone, there is an apparent concentration dependent change in the scattering curve, and the data were used without extrapolation to infinite dilution. In the case of SpeG in the presence of spermine, differences appear to match those expected from a structure factor and form-factor curves for further analysis were derived as described below.

## 2 Experimental design, materials and methods

It is common practice to remove the contribution of a concentration dependent structure factor from the small angle scattering of a more concentrated sample by extrapolating data collected on a dilution series to infinite dilution [2], [3]. These extrapolated data points are then used to replace the low *q* data (*q*<0.1 Å^−1^) of the concentrated data set. This in effect treats the scattering curve or the concentrated sample as the product of an undetermined concentration dependent structure factor and the form factor of the particle in ideal solution. Alternatively the low *q* data can be taken from the more dilute samples, where there is no evidence of a structure factor, and combined with the higher *q* data from the more concentrated sample, where the signal to noise ratio is more favorable. Both methods yield a single form factor curve to be used in analysis with a signal to noise in the low *q* data equivalent to the most dilute sample.

**Fig. 1:**
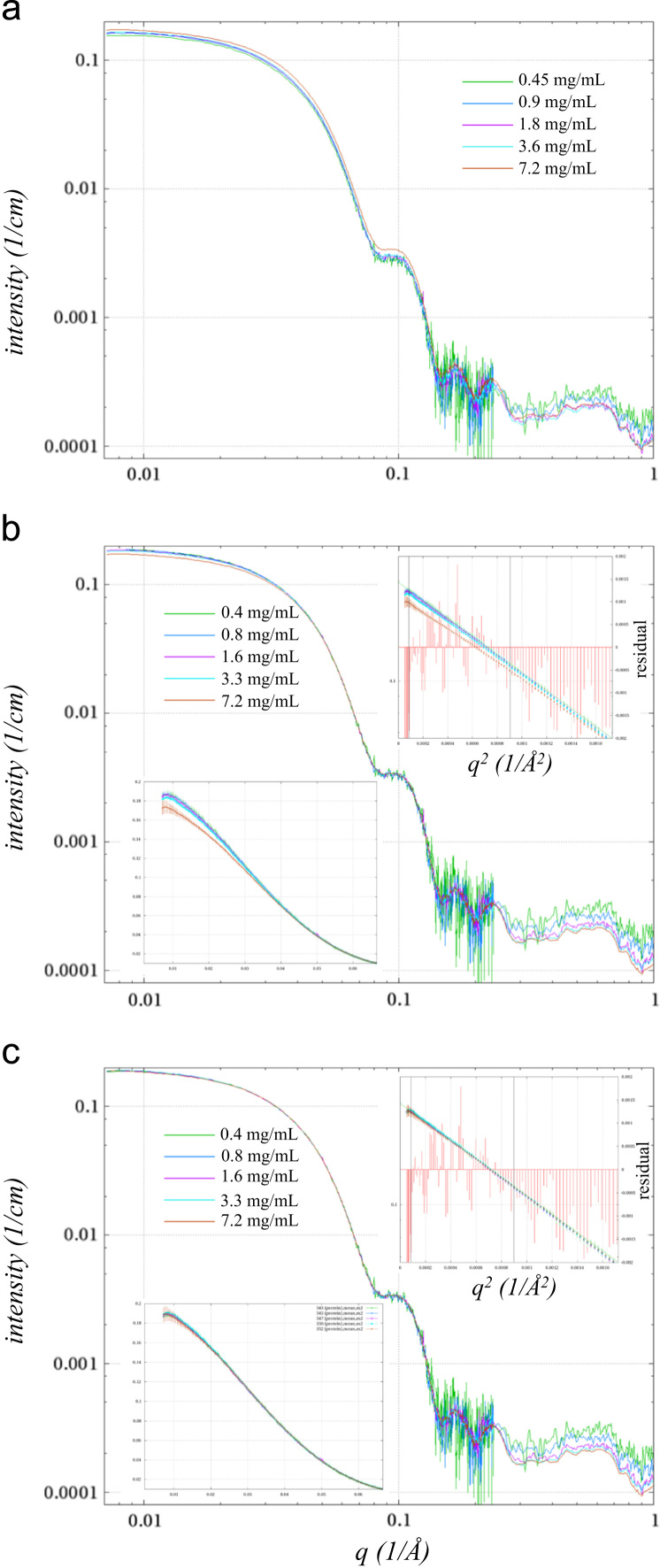
Dilution series SAXS curves from SpeG in the presence of spermine. (a) Double log plot of SAXS curves as measured with buffer and empty capillary scattering subtracted and corrected for concentration: *I*_protein_=[(*I*_sample_−*I*_empty_)−(*I*_buffer_–*I*_empty_) (1−*c*_n_ 7.4×10^−4^)]/*c*_n_, where *c*_n_ is the SpeG concentration in mg/mL as shown on the key. The given concentration is based on A_280_ of the most concentrated sample, but calculated from the 2-fold dilution for the rest of the dilutions. (b) Data concentration corrected scattering curves based on superimposition of the curves from 0.06 Å^−1^ to 0.08 Å^−1^. Upper left inset shows the Guinier plot where an *R*_g_ of 43.3 Å was derived fitting to data from 0.4/*R*_g_ to 1.3/*R*_g_ (black lines show these limits). Residuals are shown as a red impulse ordinate shown on the right. The lower left plot shows the same data plotted on linear scales to focus on the effect of the intermolecular structure factor. (c) Same plot and insets as (b), but showing the quotient of the scattering curves with the fitted structure factor divisor.

Another method for extrapolating to infinite dilution was used for the data from the SpeG solution in the presence of spermine; where a repulsive structure factor was evident as a reduction in the low *q* data, but otherwise the concentration corrected curves nearly superimposed (Fig. 1a). This method involved fitting a function representing the concentration dependent structure factor of the sample and then dividing it from each of the dilution scattering curves. By using this method a structure factor free set of data for each dilution of the SpeG in presence of spermine was calculated prior to determining the volume fractions of each oligomeric state [1]. Comparatively, for the dilution series of SpeG in the absence of spermine, a structure factor could not be calculated and hence the volume fractions of the two most concentrated samples were likely skewed to favor lower molecular weight oligomers.

To determine a concentration dependent structure factor, each curve in the dilution series first had its relative concentration scaled to correct for small pipetting errors. This was done by taking the ratio each scattering curve in the dilution series (buffer subtracted but not divided by concentration) to the scattering curve of the most concentrated sample. A horizontal line was then fit to the higher angle data between 2/*R*_g_ and 3/*R*_g_ (about 0.06 Å^−1^ and 0.08 Å^−1^) to yield a correction factor for each concentration relative to the most concentrated (Fig. 1b).

**Fig. 2:**
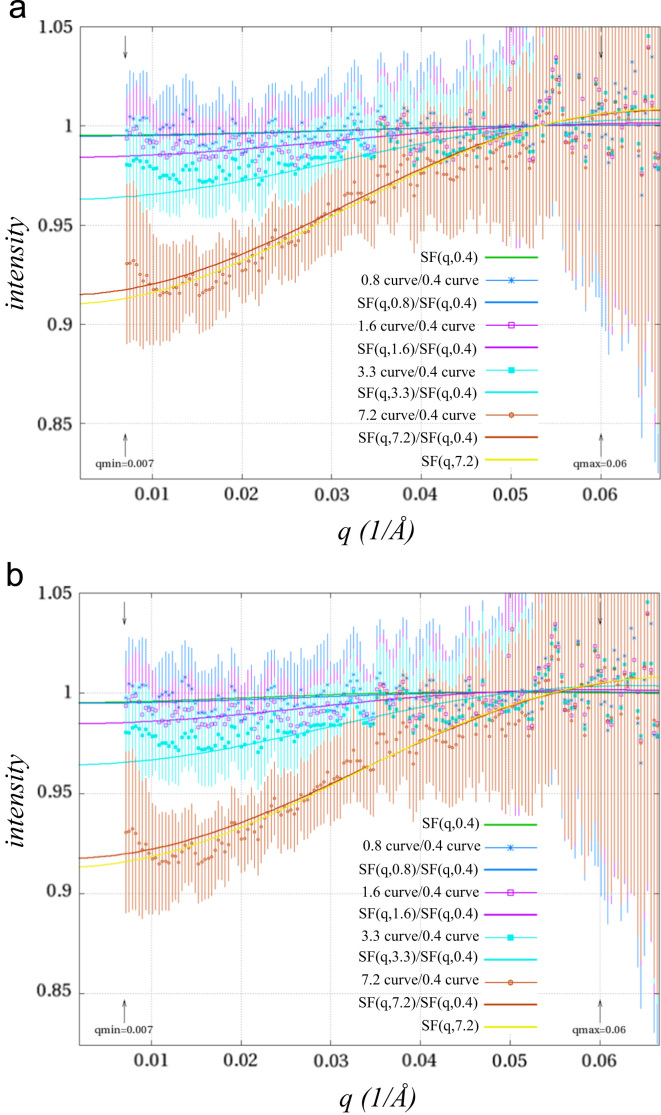
Structure factors curves from SpeG in the presence of spermine. Both plots show the measured data (as a ratio of the scattering curve from each sample in the dilution series with the most dilute sample) with points having error bars and the fitted hard-sphere structure factor equation ratios as lines of the same color. (a) Result where only *R* and *η* were varied in fitting SF(*q*,7.2) to the 7.2 curve/0.4 curve data. (b) Result after fitting all curves to SF(*q*,*c*_n_)/SF(*q*,*c*_1_) while allowing *R*_0_, *R*_m_, and *η*_0_ to vary.

**Fig. 3:**
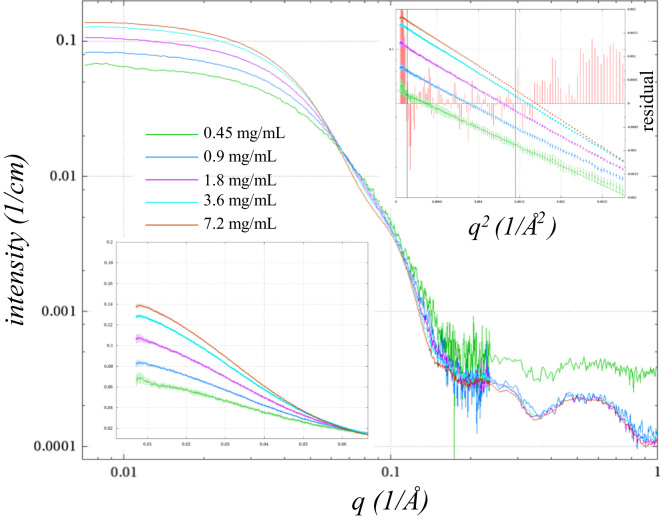
Dilution series SAXS curves from ligand-free SpeG without spermine. Double log plot of SAXS curves as measured with buffer and empty capillary scattering subtracted and corrected for concentration as in Fig. S1a. Upper left inset shows the Guinier plot where black lines show the 0.4/*R*_g_ to 1.3/*R*_g_ limits used in fitting the 34.2 Å *R*_g_ of the 0.4 mg/mL sample. Residuals are shown as a red impulse ordinate shown on the right. The lower left plot shows the same data plotted on linear scales. Since these data show clear and significant concentration dependent differences beyond that of the structure factor, neither fitting the relative concentrations, nor extrapolating to infinite dilution could be done.

Then each of the concentration corrected curves were divided by the most dilute sample (Fig. 2a) to yield structure factor curves for each of the four dilutions above the most dilute. Initially *R* (hard sphere radius) and *η* (hard sphere volume fraction) were computed using a hard-sphere form factor function [4]

(1) $$\mathrm{SF}\left(q,R,\eta \right)=\frac{1}{1+24\eta (G(2\mathrm{Rq})/2\mathrm{Rq})}$$

where

(2) $$G\left(A\right)=\frac{\left(\sin\;A-A\;\cos\;A\right)\alpha }{A^{2}}+\frac{(2A\;\sin\;A+\left(2-A^{2}\right)\cos\;A-2)\beta }{A^{3}}+\frac{(-A^{4}\;\cos\;A+4[\left(3A^{2}-6\right)\cos\;A+(A^{3}-6A)\sin\;A+6])\gamma }{A^{5}}$$

and

(3) $$\begin{array}{c} \alpha ={\left(1+2\eta \right)}^{2}/{\left(1-\eta \right)}^{4}\\ \beta =-6\eta {\left(1+\eta /2\right)}^{2}/{\left(1-\eta \right)}^{4}\\ \gamma =\eta {\left(1+2\eta \right)}^{2}/(2{\left(1-\eta \right)}^{4}) \end{array}$$

fit by nonlinear least-squares to the structure factor curve of the most concentrated sample between 0.007 Å^−1^ and 0.06 Å^−1^. This yielded an *R* of 42.4 Å and an *η* of 1.18×10^−2^ with a reduced *χ*^2^ of 0.061 (Fig. 2a). Next *η* was treated as a function of the sample concentration: *η*(*c*_n_)=*c*_n_*η*_0_; and the ratio of SF(*q*,*c*_n_)/SF(*q*,*c*_1_) was fit for four structure factors (*n*=2 to 5, where *c*_1_ is the most dilute) simultaneously giving the same results with an *R* of 42.4 Å and an *η*_0_ of 1.62×10^−3^ (*c*_5_=7.2 mg/mL) with a reduced *χ*^2^ of 0.085. Finally *R* was fit as the product of an initial value *R*_0_ and the concentration with the small fractional exponent *R*_m_: *R*(*c*_η_)=*R*_u_(*c*_η_)^R^_m_. This allows the hard sphere radius to vary according to concentration in a manner similar to that observed in other protein structure factor studies [5], [6], [7]. The final results had a reduced *χ*^2^ of 0.081 with *R*_0_=53.3 Å, *η*_0_ of 1.59×10^−3^, and *R*_m_=−0.13 (Fig. 2b). While these results are not incompatible with the possible intermolecular interactions in these samples, no physical interpretation can be assigned based on the obvious over-fitting of a simple hard-sphere model for these dilutions. Instead the fitted SF(*q*,*c*), can now applied as the divisor to the scattering curve from each dilution (*c*_1_ through *c*_5_) to produce redundant form factor data sets (Figs. 2c and 3)

## Footnotes

Appendix A Supplementary data associated with this article can be found in the online version at doi:10.1016/j.dib.2015.11.044.
